# CRISPR/Cas-based customization of pooled CRISPR libraries

**DOI:** 10.1371/journal.pone.0199473

**Published:** 2018-06-20

**Authors:** Jiyeon Kweon, Da-eun Kim, An-Hee Jang, Yongsub Kim

**Affiliations:** 1 Department of Biomedical Sciences, University of Ulsan College of Medicine, Asan Medical Center, Seoul, Republic of Korea; 2 Stem Cell Immunomodulation Research Center, University of Ulsan College of Medicine, Seoul, Republic of Korea; 3 Department of Chemistry, Seoul National University, Seoul, Republic of Korea; 4 Center for Genome Engineering, Institute for Basic Science (IBS), Seoul, Republic of Korea; National Institutes of Health, UNITED STATES

## Abstract

Pooled CRISPR libraries are widely used in high-throughput screening to study various biological processes. Various pooled CRISPR libraries have been shared for CRISPR screens and useful tools have been developed to construct researcher’s own libraries, however, many researchers are struggling to create their own pooled CRISPR libraries: it is a time-consuming, labor-intensive, and expensive process. In this study, we develop a simple method to customize conventional pooled CRISPR libraries using the CRISPR/Cas9 system. We show that conventional pooled CRISPR libraries can be modified by eliminating gRNAs that target positive genes, enabling the identification of unknown target genes in CRISPR screening. CRISPR/Cas9 system can be applied as a precise tool for customizing conventional pooled CRISPR libraries and will broaden the scope of high-throughput screening technology.

## Introduction

Various pooled libraries, such as shRNA and cDNA libraries, have been developed for high-throughput functional studies[1]. Currently, the Clustered regularly interspaced short palindromic repeat (CRISPR)-Cas9 technology for genome engineering is evolving rapidly[2] and CRISPR-based pooled libraries are widely used in high-throughput functional studies to understand various biological processes[3]. The pooled CRISPR library, comprising guide RNAs (gRNAs) targeting individual genes in the genome, is used with Cas9 or Cas9 variants in loss-of-function or gain-of-function studies[4–9]. Although several groups provide pooled CRISPR libraries via the non-profit company, Addgene, researchers still require customized libraries for their investigations[10]. Since the generation of new libraries for researcher’s own study is a time-consuming, labor-intensive, and expensive process, we developed an innovative method for customizing conventional pooled CRISPR libraries. We show that the sequence-specific depletion of desired gRNAs from conventional CRISPR libraries is possible and these gRNA-depleted libraries can be used for high-throughput screening. Furthermore, we demonstrate that 81 gRNAs, targeting 27 kinase genes related to the targets of FDA-approved drugs can be simultaneously eliminated from pooled CRISPR libraries. Based on our results, Cas9 ribonucleoproteins (RNPs) can be employed to customize conventional pooled CRISPR libraries for use in various biological screening procedures.

## Materials and methods

### Purification of Cas9 proteins and *in vitro* transcription of rc-gRNAs

Recombinant Cas9 proteins were expressed in *Escherichia coli* and purified by affinity chromatography using Ni-NTA agarose beads. LB broth (400 mL) containing 50 μg/mL kanamycin was inoculated with 4 mL of overnight-cultured BL21 (DE3) cells containing pET-His6-spCas9 plasmid DNA. When optical density at 600 nm (OD_600_) reached 0.6, Cas9 protein expression was induced by treating the cells with 0.2 mM IPTG for 16 h at 18°C. The cell pellet was harvested by centrifugation at 6,000 × g for 20 min and stored at −80°C until purification. For protein purification, the cell pellet was thawed on ice for 15 min and lysed with 4 mL of lysis buffer (50 mM NaH_2_PO_4_, 300 mM NaCl and 10 mM imidazole, pH 8.0) supplemented with 1 mM PMSF, 1 mM DTT, 1 mg/mL lysozyme and 600 units of Benzonase^®^ Nuclease. The cell lysate was cleared by centrifugation at 10,000 × g for 30 min and applied to Ni-NTA agarose beads according to the manufacturer’s instructions and an imidazole gradient (20 mM wash buffer and 250 mM elution buffer) were used to wash and elute the proteins. The eluent was buffer-exchanged with Cas9 storage buffer (20 mM HEPES, 150 mM KCl, 1 mM DTT and 40% glycerol, pH 7.5) using a PD-10 desalting column and concentrated using Amicon^®^ Ultra-4 100K according to the manufacturer’s instructions. The purified Cas9 proteins were separated by SDS-PAGE. gRNAs were synthesized *in vitro* using T7 RNA polymerase. Templates for gRNA synthesis were synthesised by annealing and extending two complementary oligonucleotides, which are listed in Table B in S1 File. For a 100-μL *in vitro* transcription reaction, T7 RNA polymerase buffer containing 1.5 μg of PCR products, each NTP at 4 mM, 14 mM MgCl_2_ and 100 units of RNase inhibitor was incubated with 500 units of T7 RNA polymerase for 3 h at 37°C. Subsequently, the reaction mixture was treated with DNase I for 30 min at 37°C and the *in vitro-*transcribed gRNAs were purified using an miRNeasy kit.

### Cas9 RNPs treatment for gRNA depletion from pooled CRISPR libraries

For generating the gRNA-depleted library, 20 μg of pooled CRISPR library plasmids DNA (human GeCKO v1 and v2 lentiviral sgRNA libraries, Addgene #1000000049) was incubated with NEBuffer 3.1 containing 1× Cas9 RNPs and a complex of 10 μg of Cas9 proteins and 7.5 μg of rc-gRNAs for 8 h at 37°C. Subsequently, the mixture was treated with RNase A for 30 min at 37°C and the gRNA-depleted libraries were purified by isopropanol precipitation.

### qPCR and targeted deep sequencing

qPCR and targeted deep sequencing were performed to assess the quality of the gRNA-depleted libraries. For qPCR, a common forward primer, annealing to the U6 promoter, and target-specific reverse primers were used with the 2× iQ^™^ SYBR^®^ Green Supermix. A primer pair specific to the puromycin gene in the plasmid DNA was used for internal control PCR amplifications. One nanogram of pooled libraries was subjected to qPCR and the comparative C_T_ method was used to estimate specific gRNA depletion. For targeted deep sequencing, PCR amplicons of gRNA-encoding regions were sequenced on MiniSeq or NextSeq system as described previously[11]. Sequencing data were analyzed using count_space.py as described previously[12].

### Cell culture and CRISPR library screening

HEK293T cells (ATCC CRL-11268) and HeLa cells (ATCC, CCL-2) were maintained in Dulbecco’s modified Eagle’s medium (DMEM) supplemented with 10% fetal bovine serum (FBS) and 1% penicillin/streptomycin. HEK293T cells (5 x 10^6^) were seeded on a 100-mm dish one day before transfection and transfected with 15 μg of the lentiviral library and two viral packaging plasmids (9 μg of psPAX2 and 6 μg of pMD2.G) using Lipofectamine 2000 according to the manufacturer’s instructions and the culture medium was changed after 6 h. The lentiviral particles were harvested and filtered using a 0.45-μm filter 48 h after transfection.

HeLa cells (1 x 10^7^) were plated on a 100-mm dish and transduced with the lentivirus (MOI ~0.1). The transduced cells were selected on media containing 1 μg/mL puromycin. For 6-TG screening, 1 μg/mL 6-TG was added to the culture medium 3 days after transduction. After two weeks, crystal violet staining was performed to visualize 6-TG-resistant clones and genomic DNA was isolated for analysis by targeted deep sequencing. The screening data was analysed using the pipeline MAGeCK (ver. 0.5.6)[13].

## Result and discussion

**Fig 1:**
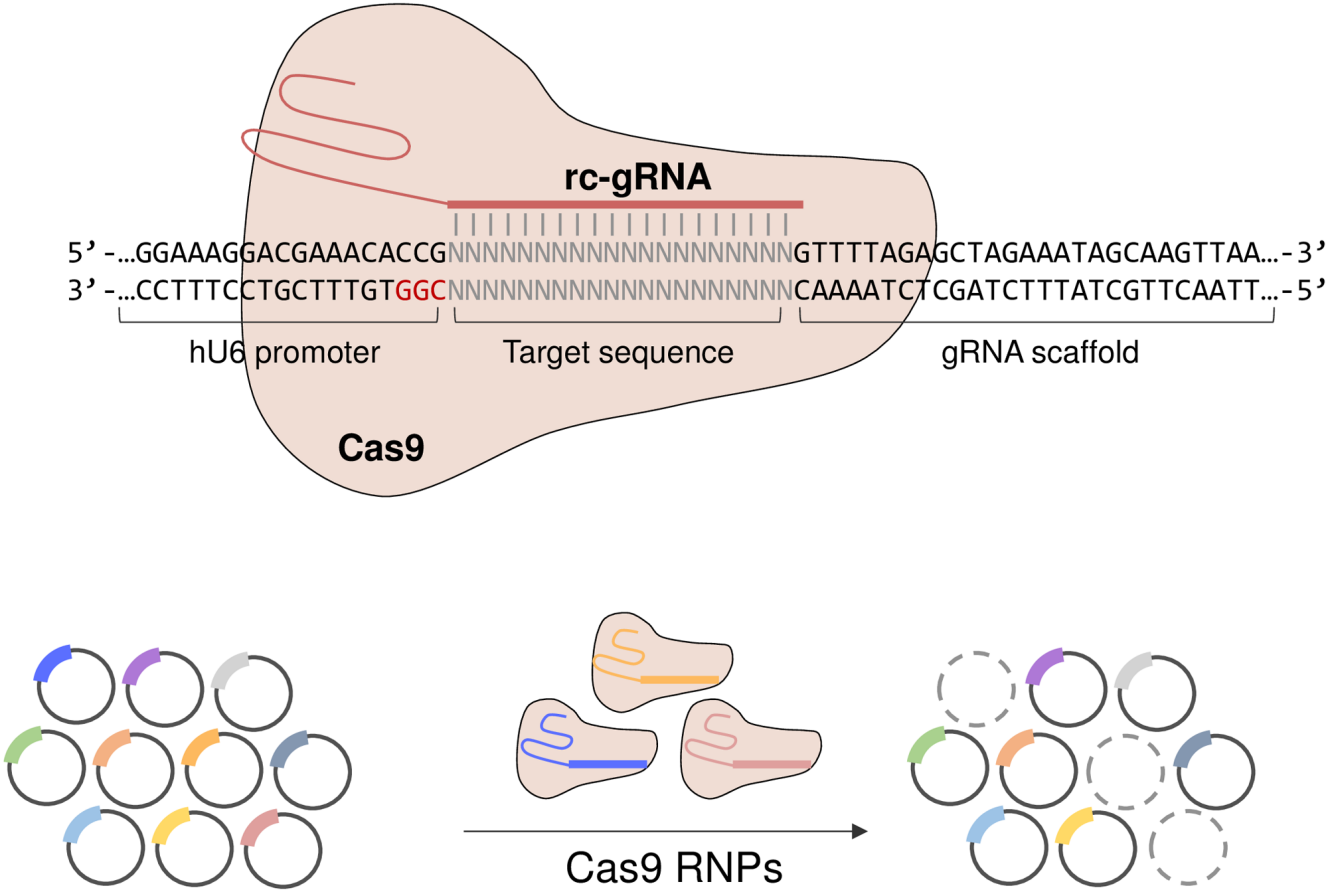
Schematic outline of customization of pooled CRISPR library. The gRNA-encoding plasmid DNA in the pooled CRISPR library contained the nucleotide sequence 5’-CGG-3’, which could be used as the PAM sequence for Cas9 proteins. Cas9 RNPs, which are complexes of Cas9 proteins and rc-gRNAs, could selectively cleave plasmid DNA possessing complementary sequences with rc-gRNA. Based on this principle, only the desired gRNAs could be depleted from the pooled CRISPR library.

We first noted that gRNAs in the pooled CRISPR library are expressed from the U6 promoter (originating from human or mouse) and the promoter contains the sequence 5′-CCG-3′ upstream of the gRNA-encoding region. Using these as PAM sequences for Cas9 proteins, reverse-complementary gRNAs (rc-gRNAs) could be designed to target each gRNA in the pooled CRISPR plasmid DNA library, which would eliminate specific gRNAs from the pooled CRISPR library (Fig 1). As proof of concept, we treated Cas9 RNPs to remove *HPRT1* gRNAs from the human GeCKOv2 library[14]. Cas9 RNPs, comprising Cas9 proteins and *in vitro*-transcribed rc-gRNAs targeting *HPRT1* gRNAs, were treated to the GeCKOv2 plasmids DNA library *in vitro*.

**Fig 2:**
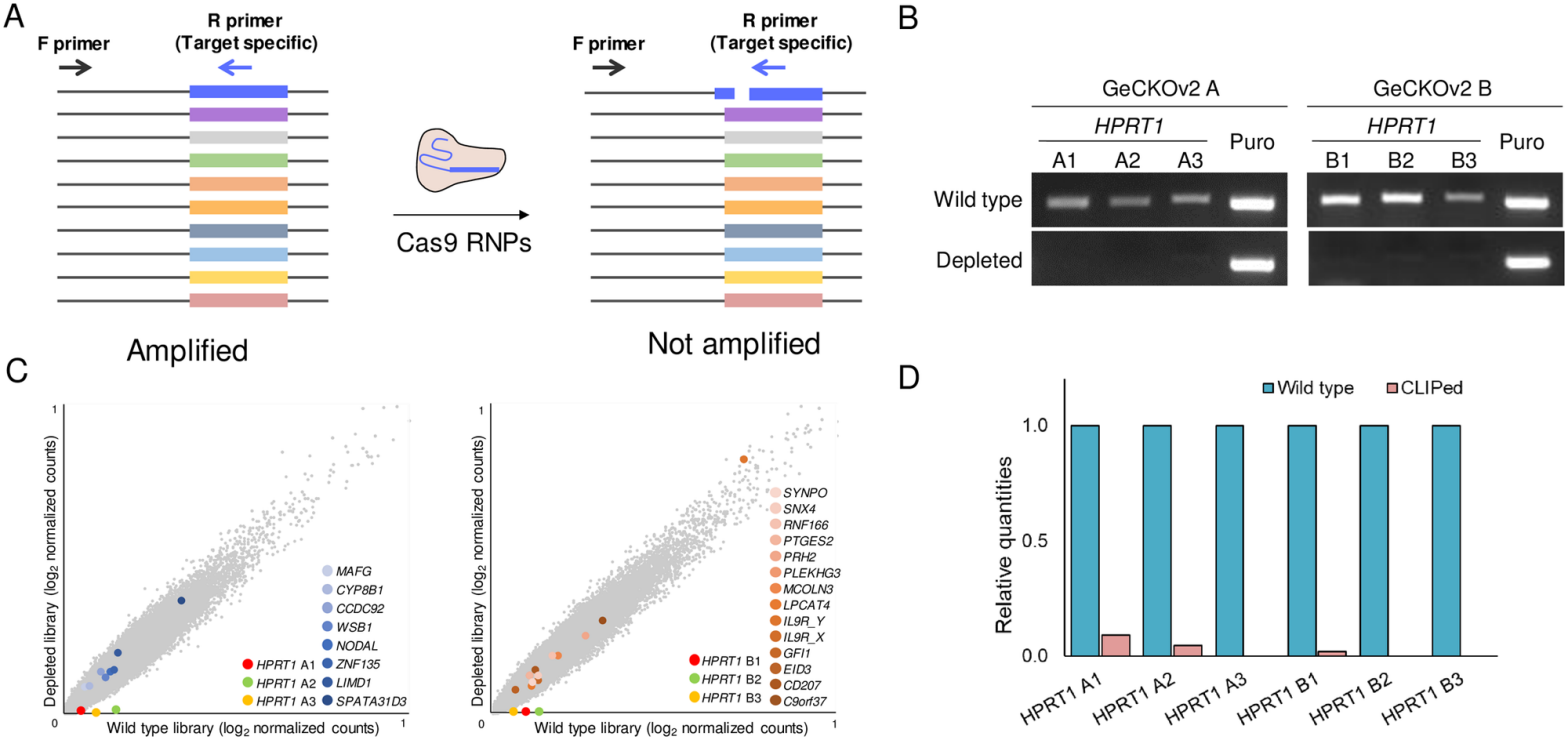
Cas9 RNPs treatment for the *HPRT1*-depleted CRISPR library generation. (A) Schematic outline of PCR to assess the quality of the gRNA-depleted library. The forward primer annealed to the U6 promoter and the reverse primers could selectively anneal to the target sequences. While using the primer pairs, amplification occurred in the wild-type library, although not in the gRNA-depleted library. (B) The PCR amplicons of the *HPRT1*-depleted GeCKOv2 library were analyzed by agarose gel electrophoresis. Each of the GeCKOv2 A and B libraries had three *HPRT1* gRNAs, all of which were depleted from each library. For all six reverse primers annealed to the *HPRT1* gRNA-encoding plasmid DNA, amplification occurred with the wild-type libraries as templates, although not with the *HPRT1*-depleted libraries. A pair of primers annealing to puromycin-encoding sequences in the plasmid DNA was used for internal control amplification. The primer sequences are listed in Table B in S1 File (C) Targeted deep sequencing analysis of the *HPRT1*-depleted libraries as described previously[11]. Scatter plots of the *HPRT1*-depleted GeCKOv2 A (left) and the *HPRT1*-depleted GeCKOv2 B (right) libraries showed that only *HPRT1* gRNAs were depleted from the wild-type GeCKOv2 libraries. (D) Relative *HPRT1* gRNA quantities were calculated from the targeted deep sequencing data. Numerical data are presented in S1 Table.

To confirm *HPRT1* gRNA cleavage, we performed polymerase chain reaction (PCR) or quantitative PCR (qPCR) using target-specific primers (Fig 2A). While using *HPRT1*-target site-specific primer pairs, PCR was conducted successfully with wild-type GeCKOv2 libraries; however, we could not detect PCR amplicons with the *HPRT1*-depleted GeCKOv2 libraries (Fig 2B and Figure A in S1 File). We confirmed that the gRNAs were depleted dose- and time-dependently (Figure B in S1 File). Additionally, we performed targeted deep sequencing to verify *HPRT1* gRNA depletion. Among 123,411 gRNAs in total (65,383 in GeCKOv2 A library and 58,028 in GeCKOv2 B library), only six *HPRT1* gRNAs were dramatically reduced in the *HPRT1*-depleted GeCKOv2 libraries (Fig 2C and 2D and S1 Table). Moreover, Cas9 RNPs were treated with another pooled CRISPR library type, GeCKOv1 for targeted gRNA depletion and the relative quantities of the gRNAs were analyzed by targeted deep sequencing; 1 to 7 gRNAs were confirmed to be successfully depleted from the wild type library (Figure C in S1 File and S2 Table), showing that Cas9 RNPs could selectively remove only plasmids of interest from pooled CRISPR libraries.

We carefully considered the potential off-target effects of Cas9 RNPs in pooled CRISPR libraries. Since all plasmid DNA in the pooled CRISPR libraries had the same PAM sequence, we assumed that the potential off-target effect could be determined only by the mismatch tolerance of rc-gRNAs. Generally, pooled CRISPR libraries are designed to minimize potential off-target effects on the genome, in order that the gRNA nucleotide sequences contained therein are not similar[4]. Additionally, more than 3-bp mismatches between gRNA and target sequence can be distinguished by Cas9 nucleases, which can inhibit the off-target effects[15]. We examined whether there were gRNAs with sequences similar to those of the six *HPRT1* gRNAs in the GeCKOv2 libraries and in fact, there was 1 gRNA with 5-bp mismatches and 21 gRNAs with 6-bp mismatches with the gRNAs (Table A in S1 File). The relative quantities of the above-mentioned gRNAs in the wild-type and the *HPRT1*-depleted libraries did not differ significantly from each other (Fig 2C and Table A in S1 File). These results showed that Cas9 RNPs could successfully eliminate specific gRNAs-encoding plasmids DNA from conventional CRISPR libraries and the potential off-target effect of Cas9 RNPs were not of concern while using well-defined pooled CRISPR libraries.

**Fig 3:**
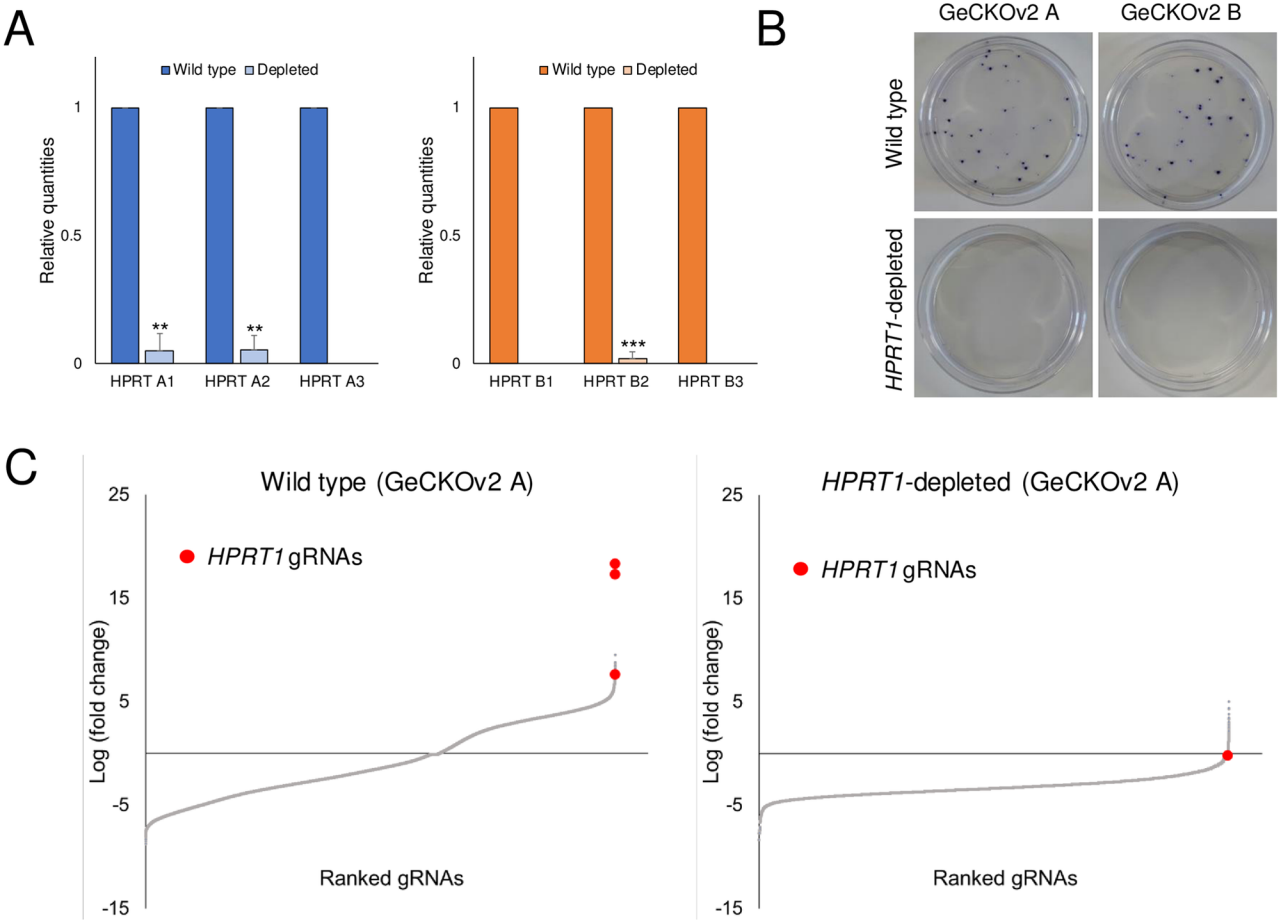
High-throughput screening with the *HPRT1*-depleted libraries. (A) Relative *HPRT1* gRNA quantities were calculated from the targeted deep sequencing data on the cells infected with each library. Error bars indicate SEM. **P < 0.01, ***P < 0.001. Student’s *t*-test. (B) Crystal violet staining images after 6-TG selection of cells infected with designated libraries. 6-TG-resistant clones were observed only in the cells infected with the wild-type libraries. (C) Log_2_-fold change in gRNAs in cells infected with the wild-type or the *HPRT1*-depleted GeCKOv2 A library after 6-TG selection. All three *HPRT1* gRNAs were highly enriched in the cells infected with the wild-type library. Numerical data are presented in S1 Table.

To determine whether the gRNA-depleted library could be used for functional studies, we performed 6-thioguanine (6-TG) screening with the wild-type and the *HPRT1*-depleted libraries. As 6-TG is cytotoxic to *HPRT1-*expressing cells, *HPRT1* gRNAs result in positive hits in 6-TG screening because they disrupt *HPRT1* and make cells resistant to 6-TG[16, 17]. Lentiviral particles were produced from the wild-type and the *HPRT1*-depleted libraries and transduced into a HeLa-Cas9 stable cell line. To measure relative quantities of gRNAs in the infected cells, genomic DNA was isolated from these cells and analyzed by targeted deep sequencing at a depth of over 80-fold. Similar to the findings on plasmid DNA, of 123,411 gRNAs, only *HPRT1* gRNAs were dramatically depleted in two independent experiments (Fig 3A and S1 Table). After confirming that the gRNAs in the cells was sufficient coverage, we conducted recessive screening with 6-TG and observed that 6-TG-resistant clones were not present in the cells infected with the *HPRT1*-depleted libraries (Fig 3B). The targeted deep sequencing analysis revealed that all *HPRT1* gRNAs were highly enriched in the cells infected with the wild-type library, although not in those infected with the *HPRT1*-depleted library, as in a previous study[17] (Fig 3C, Figure D in S1 File and S1 Table). Our results showed that Cas9 RNPs could successfully eliminate gRNAs of interest *in vitro* and the gRNA-depleted library could be used for high-throughput functional studies.

**Fig 4:**
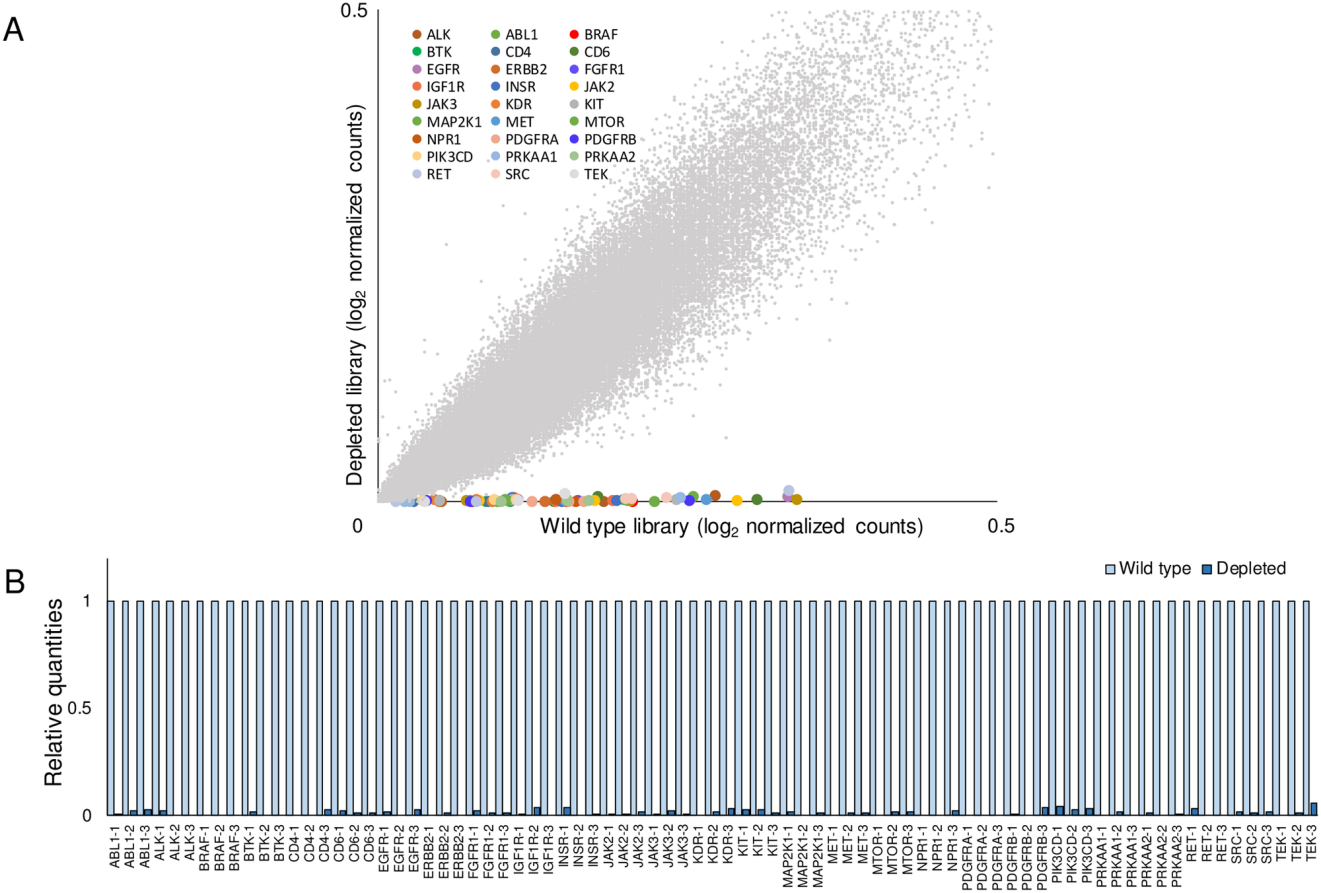
Cas9 RNPs treatment for depletion of multiple gRNAs. (A) Targeted deep sequencing analysis of the kinase-depleted GeCKOv2 A library. The scatter plots showed that all 81 gRNAs that targeted 27 human kinase genes were depleted in the kinase-depleted compared with the wild-type library. (B) Relative gRNA quantities were calculated from the deep sequencing data. Numerical data are presented in S3 Table.

Scalability of our method is essential for applications in various biological research studies. To confirm that a set of gRNAs targeting each gene could be simultaneously depleted by Cas9 RNPs, we designed rc-gRNAs for 81 gRNAs targeting 27 human kinase genes, which were known to be the targets of FDA-approved drugs[18]. Kinases are known to be potential therapeutic targets and their inhibitors are used as cancer-targeting agents[19]. However, recently, non-kinase targets of kinase inhibitors have been identified and they are gaining attention as new therapeutic targets[20]. To construct a kinase-depleted library, 81 rc-gRNAs, which were simultaneously transcribed *in vitro*, and Cas9 proteins were treated together to GeCKOv2 library A as described below in the Materials and Methods section and the quality of the kinase-depleted library was assessed by targeted deep sequencing. The relative quantities of gRNAs showed that all 81 gRNAs in the kinase-depleted library were dramatically reduced compared with those in the wild-type library (Fig 4A and 4B and S3 Table). This kinase-depleted library may be used for biomedical screening to identify new druggable target genes. Taken together, we show that Cas9 RNPs, comprising Cas9 proteins and *in vitro* transcribed rc-gRNAs, could simultaneously deplete multiple gRNAs from conventional CRISPR libraries without off-target effects and the gRNA-depleted libraries could be used for high-throughput screening.

We developed a CRISPR/Cas9 based method for customizing conventional pooled libraries. Although previous studies show that Cas9 RNP can be used for eliminating unwanted DNAs such as mitochondrial rRNA in RNA-Seq libraries[21, 22], we first showed that Cas9 RNPs could deplete only gRNAs of interest and multiple gRNAs could simultaneously be eliminated in pooled CRISPR libraries. The advantage of our method over the generation of new libraries is its ability to reduce the time to construct a pooled library (Figure E in S1 File). Furthermore, we presume this method can reduce cost and labour while removing up to 100 gRNAs (Figure F in S1 File). Interestingly, the deep sequencing results showed *HPRT1* gRNAs remained in the *HPRT1*-depleted library at low frequencies, but the *HPRT1* gRNAs were not enriched after 6-TG screenings using the *HPRT1*-depleted library. Based on these results, it appeared that the *HPRT1* gRNAs, interpreted as remaining in the *HPRT1*-depleted libraries, were either due to deep sequencing errors or did not exceed the threshold that could affect library screening.

Our method can be applied to genetic screening in diverse ways, for example, to exclude positive genes or gene sets in recessive screening for reducing the number of genetic screening rounds required to identify unknown target genes. Other types of Cas proteins which have different types of PAM sequences, such as Cpf1 with the T-rich PAM sequences, can be adopted to extend the usefulness of the method. Furthermore, this method is applicable to other library types, such as shRNA and cDNA libraries, with the potential to expand the availability of various pooled libraries in biomedical research.

## Supporting information

S1 File Supplementary figures and tables. (DOCX)

S1 Table Deep sequencing results of the *HPRT1*-depleted library. (XLSX)

S2 Table Deep sequencing results of the gRNA-depleted library (GeCKOv1). (XLSX)

S3 Table Deep sequencing results of the 27 kinases-depleted library. (XLSX)
