# Supplementary material for: CRISPR/Cas-based customization of pooled CRISPR libraries
Source: PLoS One. 2018 Jun 20;13(6):e0199473. doi: 10.1371/journal.pone.0199473 (PMC6010251; doi:10.1371/journal.pone.0199473)
Supplement: S1 File — (DOCX) [file pone.0199473.s001.docx]

**CRISPR/Cas-based customization of pooled CRISPR libraries**

Jiyeon Kweon^1,2¶^, Da-eun Kim^3,4¶^, An-Hee Jang^1,2^, and Yongsub Kim^1,2 *^

^1^Department of Biomedical Sciences, University of Ulsan College of Medicine, Asan Medical Center, Seoul, Republic of Korea

^2^Stem Cell Immunomodulation Research Center, University of Ulsan College of Medicine, Seoul, Republic of Korea

^3^Department of Chemistry, Seoul National University, Seoul, Republic of Korea.

^4^Center for Genome Engineering, Institute for Basic Science (IBS), Seoul, Republic of Korea.

^*^Corresponding author

E-mail: yongsub1.kim@gmail.com

^¶^These authors contributed equally to this work.

**
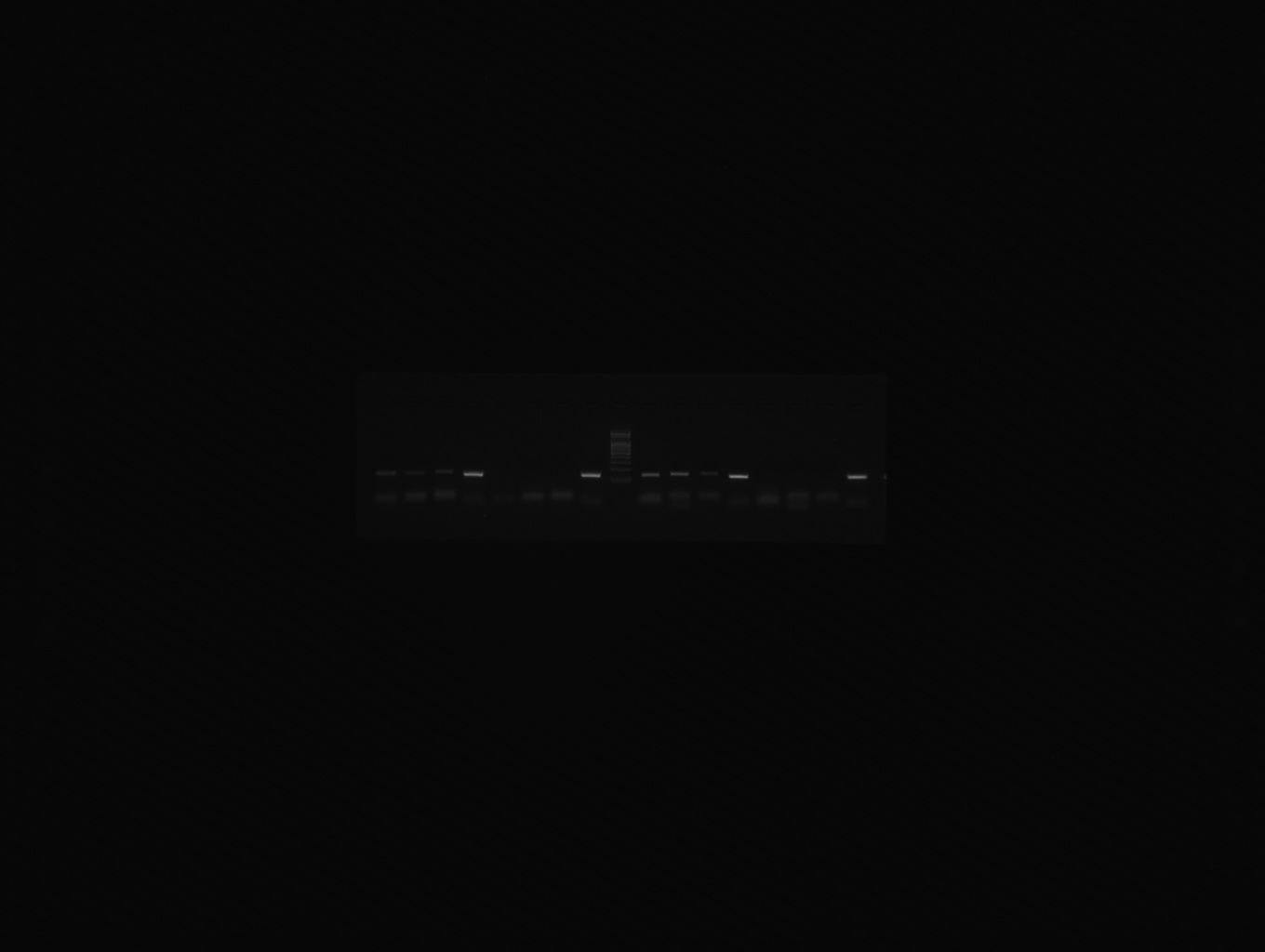
**

**
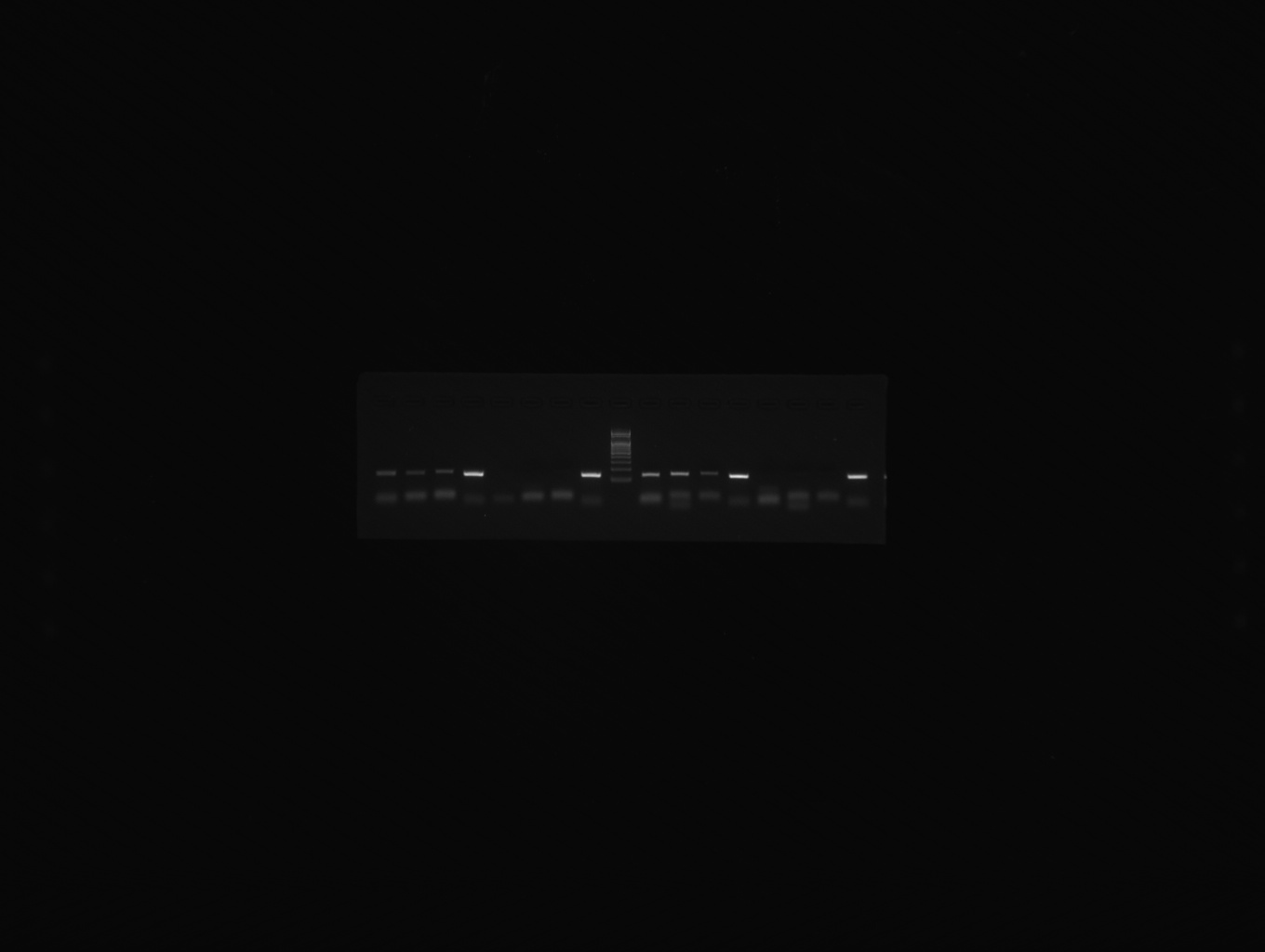
**

**Figure A. Full-length gels with low and high exposure for Figure 2b**

**A**

**
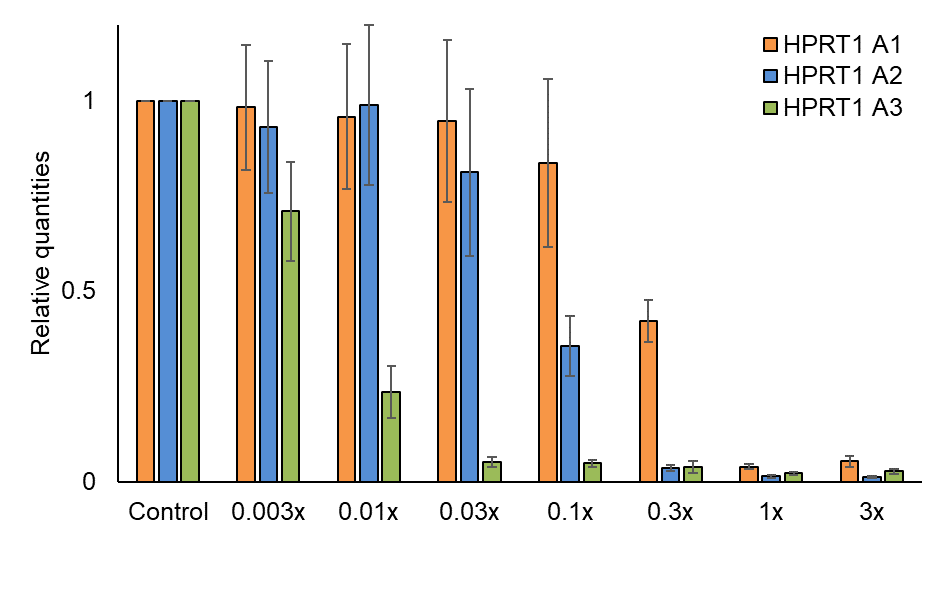
**

**B**

**
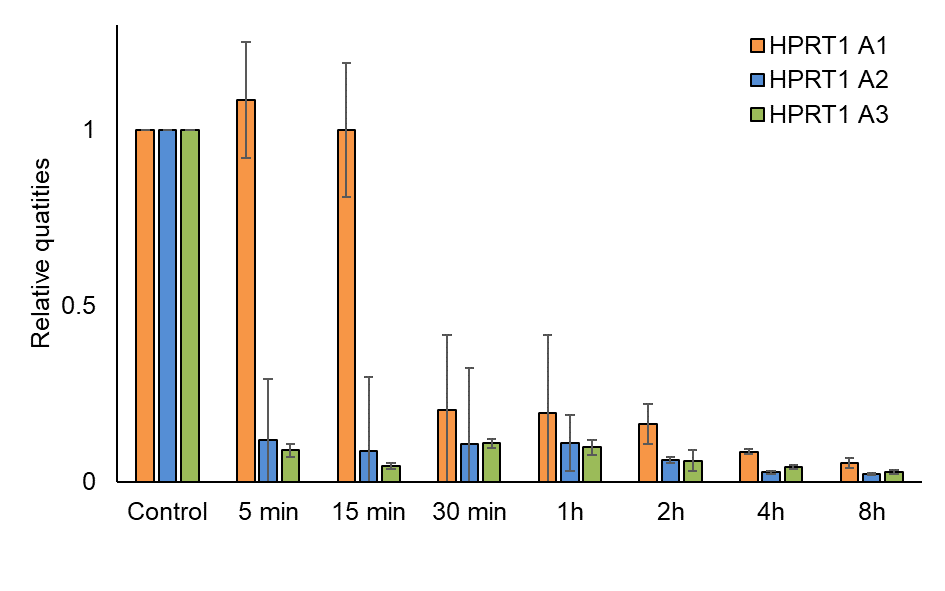
**

**Figure B. The optimization of Cas9 RNPs treatment.** (A) Serially diluted Cas9 RNPs, which are complexes of Cas9 proteins and HPRT1 rc-gRNAs, were treated to the GeCKOv2 library A for 4 h. (B) 1× Cas9 RNPs were incubated for the specified time. Relative quantities were measured by qPCR. Primer sequences are listed in Table C in S1 File. n = 3. Error bars indicate s.e.m.

**
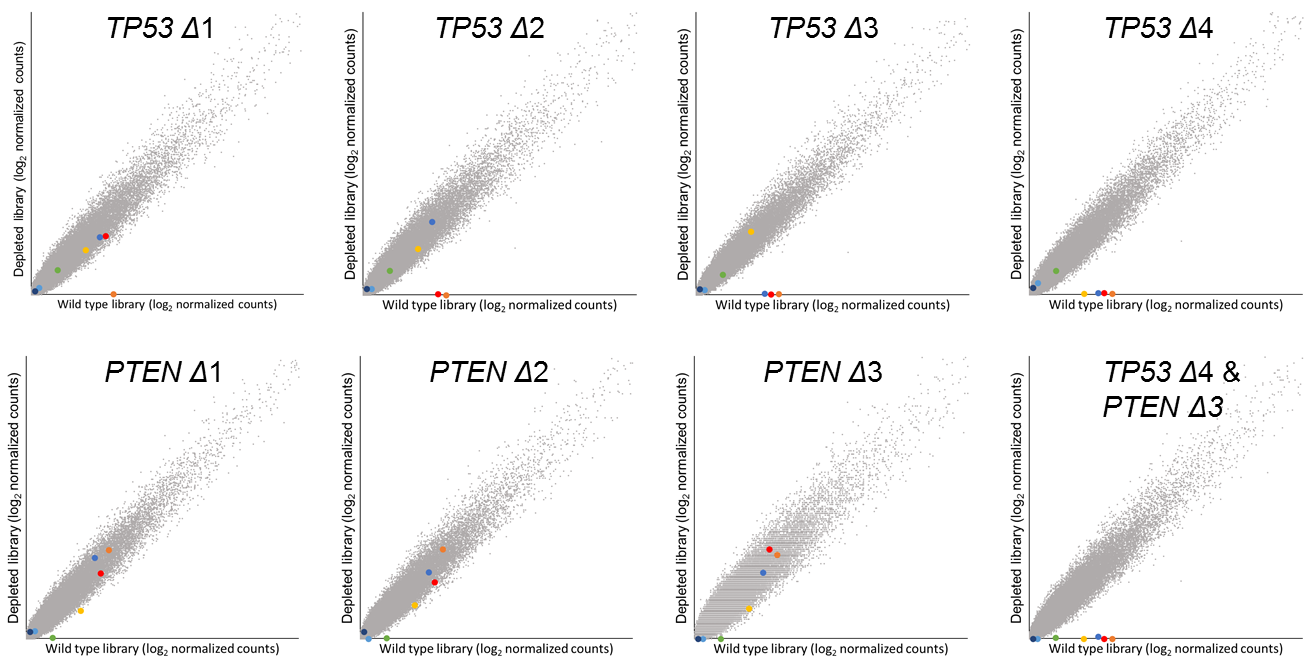
**

**Figure C. Deep sequencing analysis of gRNA-depleted GeCKOv1 libraries.** Scatter plots showed that only a specific number of gRNAs was depleted from the GeCKOv1 library.

**
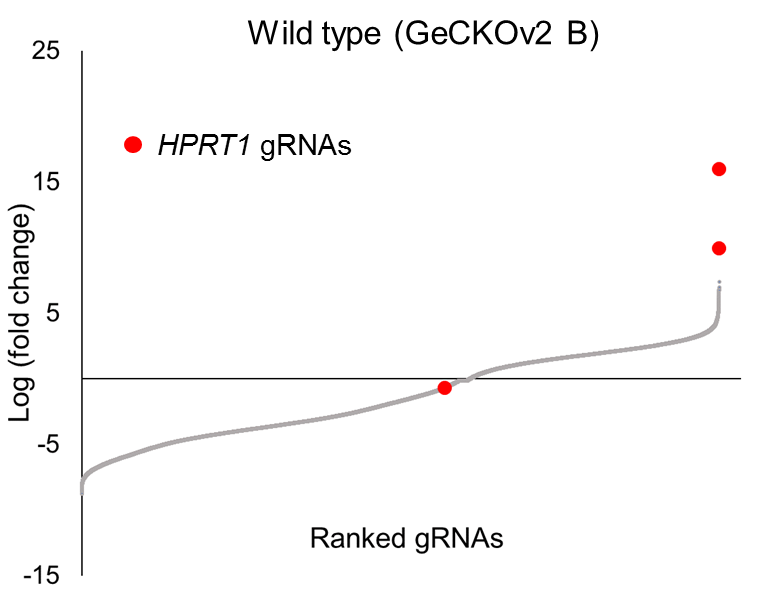
**

**
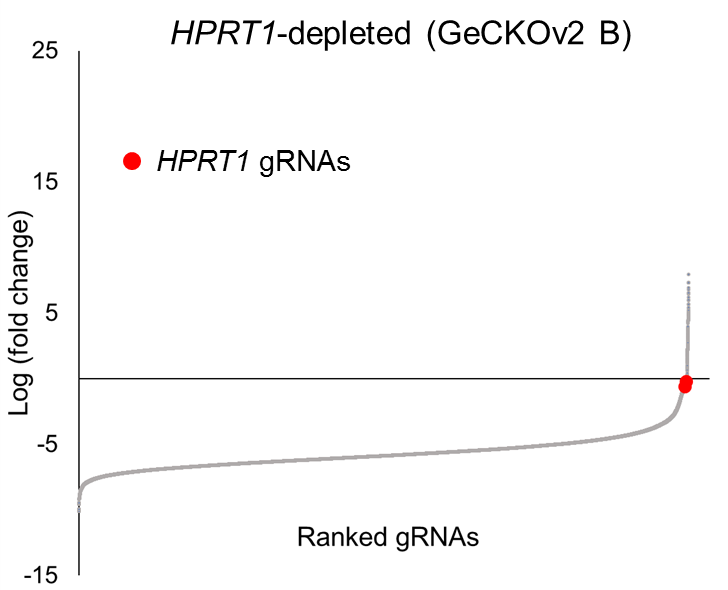
**

**Figure D. The sgRNAs were ranked by their differential abundance.** Log_2_-fold change in gRNAs in cells infected with the wild-type or the *HPRT1*-depleted GeCKOv2 B library after 6-TG selection. All three *HPRT1* gRNAs were highly enriched in the cells infected with the wild-type library. Numerical data are presented in Table A in S1 File.

**
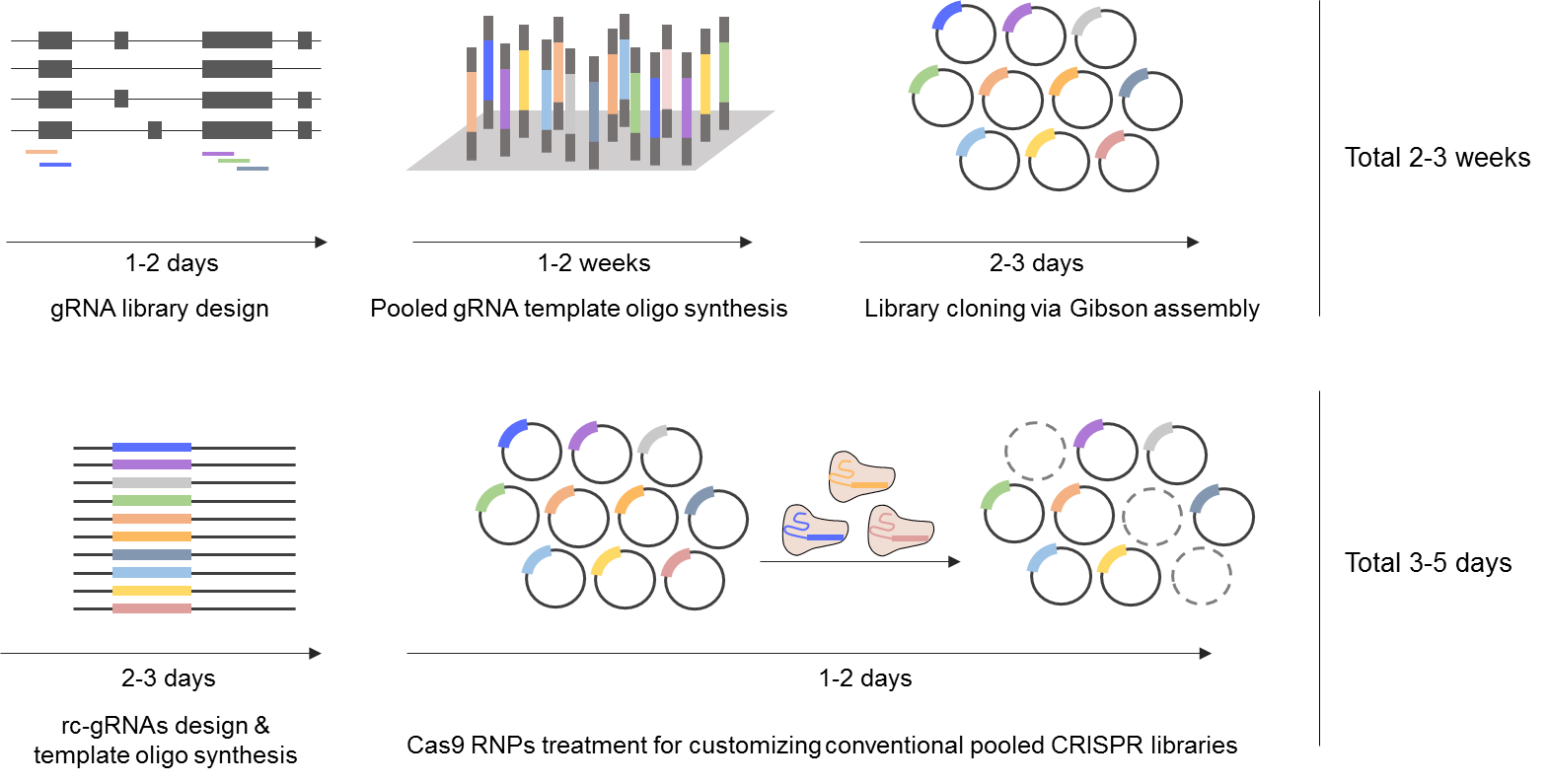
**

**Figure E. Schematic outline of comparison between conventional methods and CRISPR/Cas9-based customization of pooled CRISPR library.** Upper, conventional methods; lower, Cas9 RNPs treatment. It required 2–3 weeks to construct a pooled CRISPR library; however, Cas9 RNPs treatment could reduce the duration to 3–5 days.

**
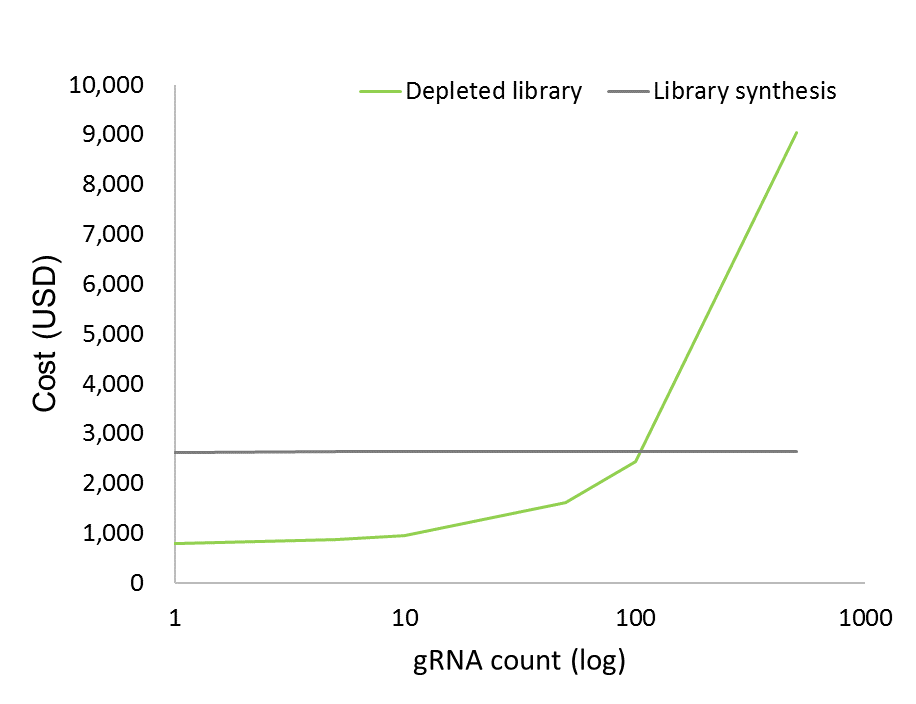
**

**Figure F. Comparison of cost between conventional methods and CRISPR/Cas9-based customization of pooled CRISPR library.** Cas9 RNPs treatment has the advantage of reducing cost and labour in removing up to 100 gRNAs.

**Table A. Potential off-target analysis of *HPRT1*-depleted libraries.**

**Table B. List of oligonucleotides for rc-gRNA synthesis.**

**Table C. List of primers for PCR.**
